# Longitudinal changes in extent of late gadolinium enhancement in repaired Tetralogy of Fallot: a retrospective analysis of serial CMRs

**DOI:** 10.1186/s12968-021-00772-x

**Published:** 2021-06-21

**Authors:** Kwannapas Saengsin, Minmin Lu, Lynn Sleeper, Tal Geva, Ashwin Prakash

**Affiliations:** 1 Department of Cardiology, Boston Children’s Hospital, 300 Longwood Avenue, Boston, MA 02115 USA; 2 Department of Pediatrics, Harvard Medical School, Boston, MA USA

**Keywords:** Tetralogy of Fallot, Late gadolinium enhancement, Cardiovascular magnetic resonance, Longitudinal study

## Abstract

**Background:**

Right ventricular (RV) late gadolinium enhancement (LGE) occurs due to surgical scarring and RV remodeling, and has been shown to be associated with clinical outcomes in Tetralogy of Fallot (TOF). However, it is not known if cardiovascular magnetic resonance (CMR) LGE extent progresses over time, and therefore, it is not known if serial reassessment of LGE is necessary. We determined the rate of progression in the extent of RV LGE on serial CMR examinations in repaired TOF.

**Methods:**

Retrospective review of 127 patients after TOF repair (49% male, median age at first CMR 18.9 years (Interquartile range (IQR) 13.3,27.0) who had at least two CMRs (median follow-up duration of 4.0 years (IQR 2.1,5.9)) was performed. 84/127 patients had no interventions between serial CMRs (Group 1) while 43/127 patients had transcatheter or surgical intervention between CMRs (Group 2). The extent of RV LGE was assessed using 2 methods: a semiquantitative RV LGE score and a quantitative RV LGE extent expressed as % of RV mass. Mixed effects linear regression modeling to estimate changes in LGE over time.

**Results:**

RV LGE was present in all patients on the first CMR. % RV LGE extent and LGE score did not increase over time in either patient group. The mean 5 year rates of change were small and negative for both % RV LGE extent [− 2.3 (95% CI − 2.9, − 1.8, *p* < 0.001) in Group 1, and − 1.9 (95% CI − 3.2, − 0.7, *p* = 0.004) in Group 2], and RV LGE score [− 0.9 (95% CI − 1.1, − 0.6, *p* < 0.001) in Group 1, and − 0.5 (95% CI − 1.1, − 0.0, *p* = 0.047) in Group 2].

**Conclusions:**

In this serial CMR evaluation of children and adults with repaired TOF, no significant progression in the extent of RV LGE was seen on intermediate term follow-up. Given recent concerns regarding the safety of gadolinium-based contrast agents, frequent assessment of LGE may not be necessary in follow-up.

## Background

Despite advances in the surgical, transcatheter, and medical management of Tetralogy of Fallot (TOF), late morbidity and mortality remain unacceptably high, related to chronic pulmonary regurgitation, ventricular dysfunction, and ventricular or atrial arrhythmias [1–5]. Right ventricular (RV) late gadolinium enhancement (LGE) on cardiovascular magnetic resonance (CMR) study can occur due to surgical scarring or RV remodeling and has been associated with ventricular dysfunction, arrhythmia, and serum biomarkers of heart failure [6–15]. Although the prognostic significance of LGE in TOF has been previously documented, data regarding LGE progression over time are sparse [16]. Understanding the rate of progression in RV LGE is important in informing the appropriate frequency of gadolinium administration to assess LGE on follow-up CMRs. This is particularly important in light of recent reports of long-term gadolinium deposits in the brain and other organs [17, 18]. The purpose of this study was to examine serial CMR data to determine the rate of progression in RV LGE after TOF repair.

## Methods

### Subjects

A retrospective review of all patients with repaired TOF who had undergone 2 or more CMR examinations with LGE imaging between January 2005 and November 2019 was performed. Patients with pulmonary atresia and major aortopulmonary collateral arteries and those with significant imaging artifacts were excluded. Demographic and clinical data were abstracted from electronic medical records. The hospital’s Committee on Clinical Investigation waived the requirement for informed consent.

### CMR

All CMR examinations were performed using a whole-body 1.5 T CMR scanner (Achieva, Philips Healthcare, Best, The Netherlands) using standard imaging techniques recommended by the Society for Cardiovascular Magnetic Resonance [19]. Ventricular volumes and ejection fraction, and pulmonary regurgitation fraction were calculated using standard techniques as previously described by our laboratory [20, 21]. LGE imaging was performed 10–15 min after intravenous administration of a gadolinium-based contrast agent (0.15–0.2 mmol/kg of gadopentate dimeglumine or gadobutrol) using a 2-D phase-sensitive inversion-recovery prepared, fast-gradient echo sequence in ventricular long- and short-axis planes using the following imaging parameters: slice thickness 6–8 mm, slice gap 0–2 mm, reconstructed in-plane spatial resolution between 1.4 × 1.4 mm and 1.7 × 1.7 mm. The short-axis imaging planes were prescribed to cover both ventricles from base to apex. Image analyses were performed using commercially available software (cvi42, v 5.10.1, Circle Cardiovascular Imaging, Calgary, Alberta, Canada).

### LGE assessment

**Fig. 1:**
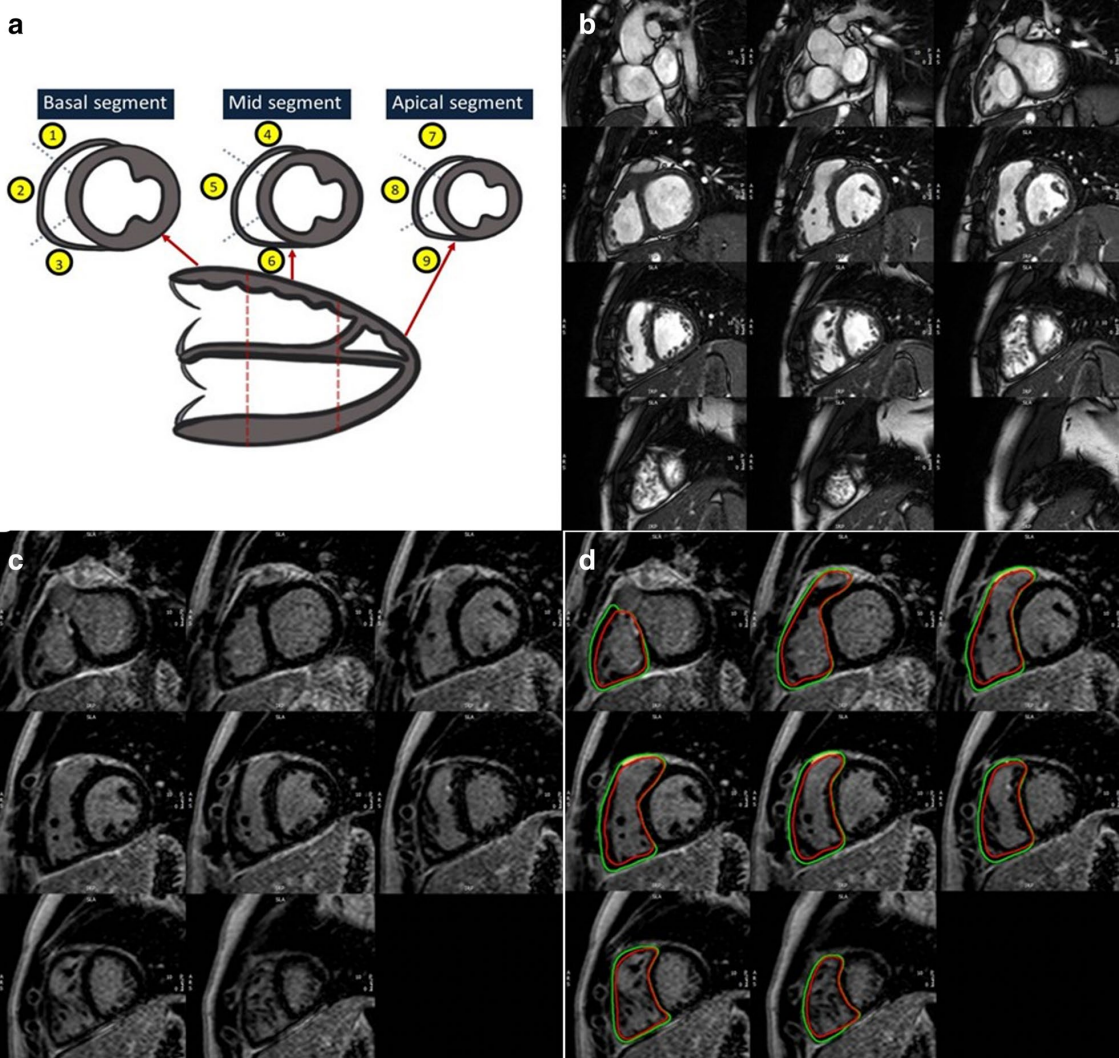
RV LGE Assessment. **a** RV LGE score: RV divided into 9 segments. Each segment graded as follows: 0—no LGE; 1—LGE involving < 1/3 of segment; 2—LGE involving 1/3—2/3 of segment; 3—LGE involving $>$ 2/3 of segment (maximum possible score 27) **b** Example of cine ventricular short axis in diastolic phase **c** Example of LGE imaging in the ventricular short axis. **d** % RV LGE Extent: Areas of LGE highlighted using semi- automated manual thresholding. % RV LGE Extent = (RV LGE mass/ Total RV mass) × 100

The extent of RV LGE was determined using 2 techniques (Fig. 1): a semiquantitative RV LGE score, and quantitative RV LGE extent expressed as % of RV mass as follows:

- **Semiquantitative RV LGE score:** The score is a modification of a previously described technique [8]. The RV was divided into 9 segments as previously described [7]. Briefly, the RV was first divided into 3 longitudinal regions—basal, midventricular, and apical. Each longitudinal segment was then divided into 3 parts—superior, anterior, and inferior, yielding a total of 9 segments. For each RV segment, LGE extent was graded by a single-blinded observer using a semiquantitative scale as follows: 0 = No LGE; 1 = LGE involving < 1/3 of the segment; 2 = LGE involving 1/3 to 2/3 of the segment; 3 = LGE involving > 2/3 of the segment (Fig. 1). The maximum possible RV LGE score for each subject was 27. Septal insertion point LGE was excluded from RV LGE for this analysis.
- **Quantitative RV LGE Extent (%):** RV endocardial and epicardial contours were drawn manually on the stack of short-axis LGE images, avoiding epicardial fat. Areas of LGE were identified visually and then a semi-automated thresholding technique was applied until the visually identified areas of LGE were marked as areas of positive LGE (Fig. 1). Percentage RV LGE extent was calculated as a sum of the RV mass exhibiting positive LGE, expressed as a percentage of total RV mass. Septal insertion point LGE was excluded from RV LGE for this analysis.

### Statistical analysis

Comparisons among independent groups were performed using Student’s t-test, analysis of variance, Wilcoxon rank-sum test or Kruskal–Wallis test, or a Fisher exact test, as appropriate. Spearman correlation was used to estimate the magnitude of association between LGE score and % LGE extent. To evaluate whether change scores in CMR outcomes differed from zero, a one-sample t-test and Wilcoxon signed-rank test were used. Mixed effects linear regression (fixed time, random subject) was used to evaluate the rate of change in RV LGE over time. For this study, assuming a standard deviation of 2.0 for the change in RV LGE score between 2 CMRs and a 2-sided 0.05-level test, with 40 subjects in each of 2 independent groups, there is 80% power to detect a mean group difference of 1.0 between the mean RV LGE change scores. Intra-rater and inter-rater reliability were assessed using the intra-class correlation coefficient (ICC). A p-value of less than 0.05 was considered significant. Analyses were performed using SAS (version 9.4, SAS Institute, Inc., Cary, North Carolina, USA) and R (version 3.5.1, R Foundation for Statistical Computing, Vienna, Austria).

## Results

### Subjects

Patient characteristics are summarized in Table 1. A total of 269 CMR examinations were analyzed on 127 subjects with a median follow-up duration of 4.0 years (IQR 2.1,5.9) between initial and latest CMR examinations. Among the study cohort, 112 subjects (88%) had 2 CMR examinations, and 15 (12%) had 3 CMR examinations available for analysis. The study sample consisted of children and adults, a majority of whom had a transannular patch during their initial surgical repair, which was performed most commonly during the first year of life. 84/127 subjects had no interventions between serial CMRs (Group 1), while 43/127 subjects had transcatheter or surgical intervention between CMRs (Group 2). The types of interventions performed in Group 2 are summarized in Table 1.

**Table 1:** Patient Characteristics on Initial CMR

	Group 1 (n = 84)	Group 2 (n = 43)	*p*-value
Male	41 (48.8%)	26 (60.5%)	0.261
Age at repair (years)	0.7 (0.3, 5.0)	0.5 (0.3, 2.9)	0.507
Type of repair			0.184
Valve sparing	15 (18.9%)	2 (4.7%)	
Transannular patch	52 (61.9%)	33 (76.7%)	
RV-to-PA conduit	7 (8.3%)	3 (7.0%)	
Unknown	10 (11.9%)	5 (11.6%)	
Time since repair (years)	18.9 (13.3, 27.0)	14.8 (12.7, 27.8)	0.179
Age at first CMR, years	20.4 (13.2, 34.2)	15.6 (13.0, 28.7)	0.164
Age at last CMR, years	25.6 (17.8, 39.6)	19.9 (12.6, 31.8)	0.067
Type of intervention between CMRs			N/A
Surgical pulmonary valve replacement	N/A	37 (86%)	
Transcatheter pulmonary valve replacement	N/A	6 (14%)	

Variables are expressed as either frequency (%) or median (interquartile range). Group 1—patients with no interventions between serial CMRs; Group 2—patients with transcatheter or surgical interventions between CMRs. *CMR* cardiovascular magnetic resonance; *RV-to-PA* right ventricle to pulmonary artery. *N/A* not applicable

### RV LGE presence, location, and extent

RV LGE was present in all patients on the first CMR examination in both groups. The location of LGE was a basal superior segment in 127 subjects (100%), at the mid superior segment in 82 (65%), and at the mid anterior segment in 16 subjects (13%) (Fig. 3). There was good correlation between RV LGE score and % RV LGE extent in both groups (r = 0.63 in Group 1, r = 0.80 in Group 2; *p* < 0.001 for both). Intra-observer reliability in measuring % RV LGE extent was good (ICC 0.83) in a subset of 40 CMR examinations that were reanalyzed at a later date by the same blinded observer (KS). Inter-observer reliability was modest (ICC 0.6), assessed in a subset of 20 CMR examinations by a blinded second observer (AP).

### Serial changes in RV LGE

**Fig. 2:**
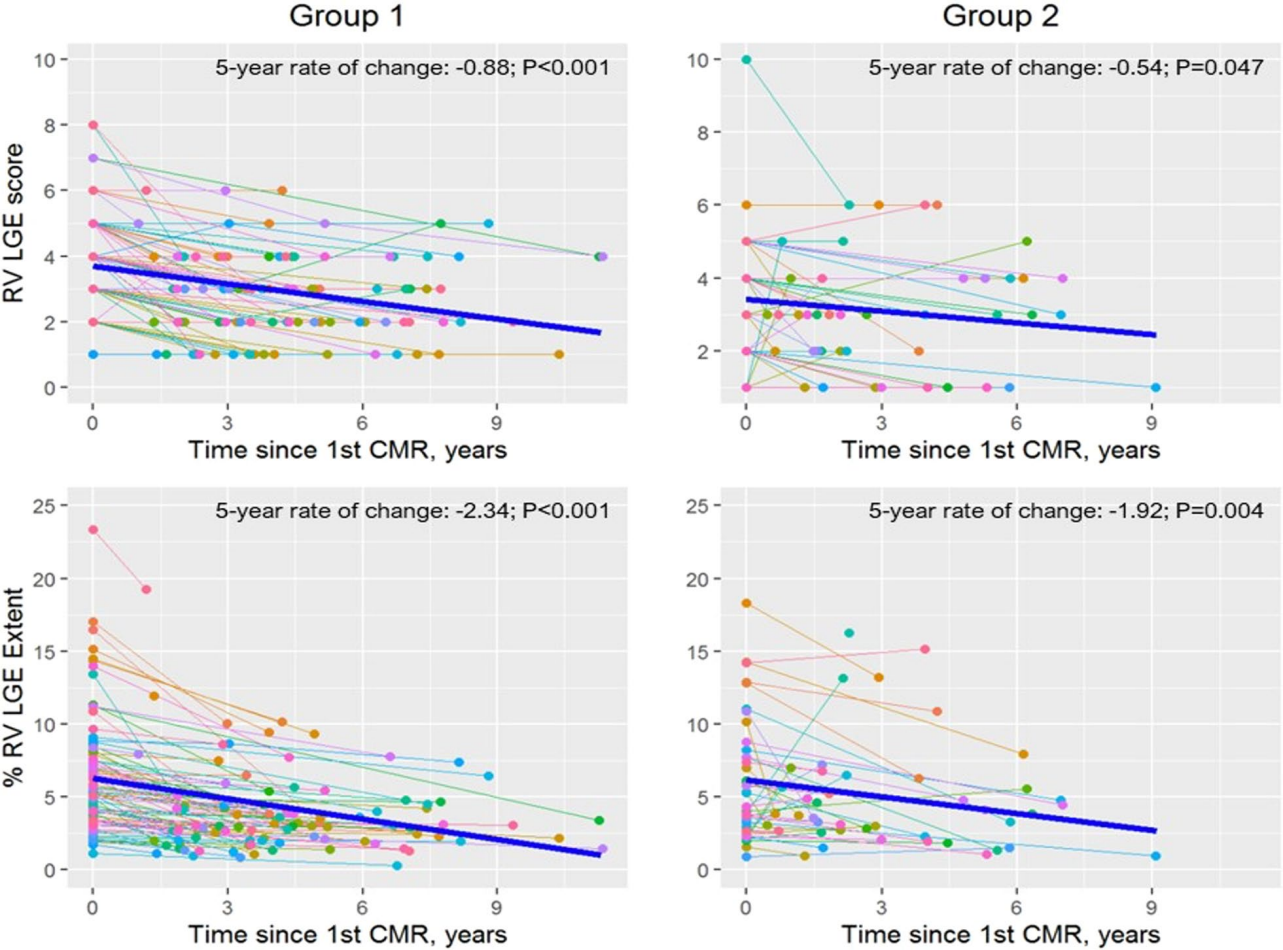
Longitudinal Changes in RV LGE Score and % RV LGE Extent. Group 1—patients with no interventions between serial CMRs; Group 2—patients with transcatheter or surgical interventions between CMRs. The thick blue line represents the estimated slope of change over time determined using linear mixed-effects modeling

**Fig. 3:**
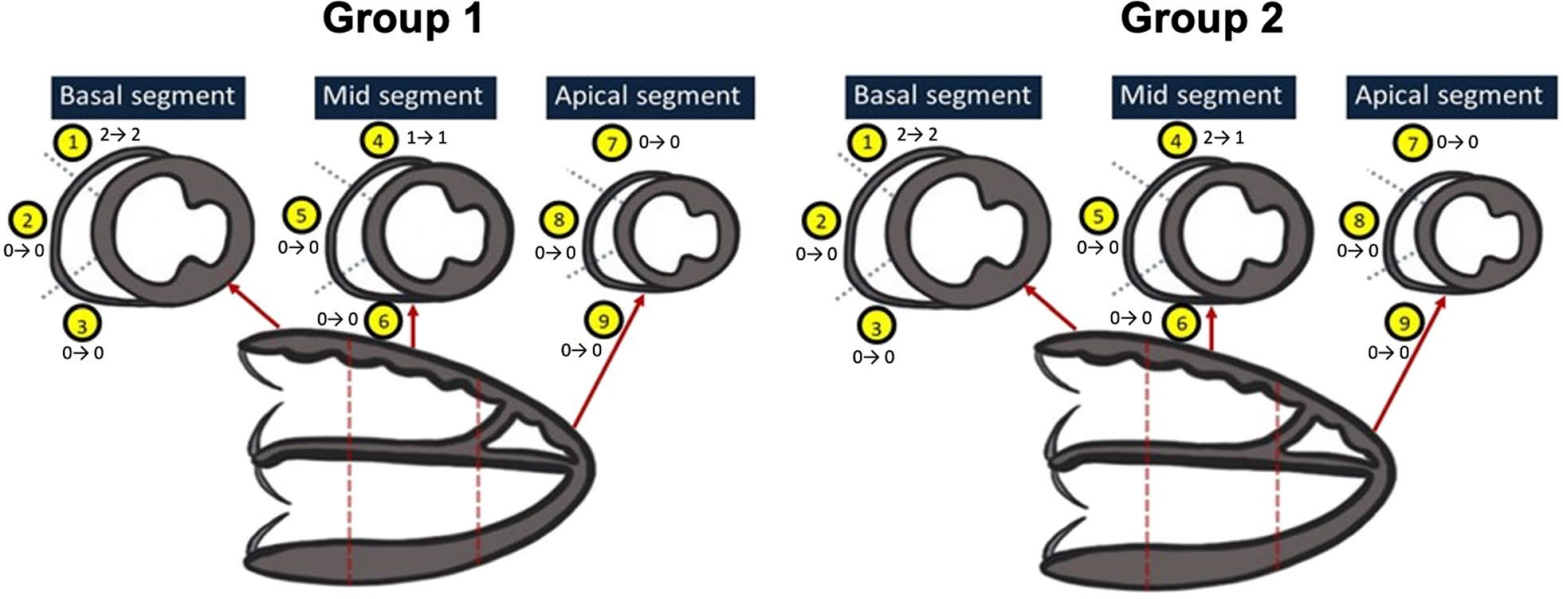
Longitudinal Changes in Segmental RV LGE Score. Group 1—patients with no interventions between serial CMRs; Group 2—patients with transcatheter or surgical interventions between CMRs. Median baseline and follow-up RV LGE scores for each segment are annotated next to each segment

Serial changes in RV LGE score and % RV LGE extent are summarized in Tables 2 and 3 and in Figs. 2 and 3. There was no increase in RV LGE over time, with a small decline in both LGE parameters over time in both patient groups. Mean 5 yeaar changes in both parameters from linear mixed-effects models are summarized in Table 3. As seen in Fig. 2, RV LGE score and % RV LGE extent increased over follow-up in a small minority of patients, and remained stationary or declined in most patients.

**Table 2:** Serial Changes in RV LGE and Functional Parameters

	Initial CMR	Latest CMR	*p*-value
Group 1 (n = 84)			
RVEDVI (ml/m^2^)	134.5 (114.0, 150.0)	133.9 (117.1, 152.1)	0.013
RVESVI (ml/ m^2^)	65.7 (55.1, 81.3)	72.0 (60.8, 84.9)	< 0.001
RVEF (%)	49.3 (45.8, 54.8)	47.6 (41.4, 51.5)	< 0.001
PR fraction (%)	29.0 (7.0, 40.0)	33.0 (9.0, 43.0)	0.002
RV LGE score	4 (3, 5)	3 (2,4)	< 0.001
RV LGE extent (%)	5.5 (3.4, 8.0)	3.1 (2.0, 4.6)	< 0.001
RV Mass/BSA, g/m^2^	16.6 (14.0, 19.4)	16.6 (14.7, 19.0)	0.652
RV Mass:Volume, g/ml	0.12 (0.10, 0.16)	0.12 (0.10, 0.15)	0.318
Group 2 (n = 43)			
RVEDVI (ml/m^2^)	175.7 (141.6, 200.4)	118.7 (102.2, 144.3)	< 0.001
RVESVI (ml/ m^2^)	85.6 (67.8, 106.1)	63.8 (51.5, 82.8)	< 0.001
RVEF (%)	50.1 (42.7, 55.0)	46.0 (40.7, 50.5)	0.013
PR fraction (%)	46.0 (30.0, 55.0)	5.0 (0.0, 13.0)	< 0.001
RV LGE score	4 (2, 5)	3 (2, 4)	0.030
RV LGE extent (%)	4.4 (2.7, 7.7)	3.9 (2.6, 6.3)	0.009
RV Mass/BSA, g/m^2^	19.1 (14.9, 22.5)	14.7 (12.5, 16.8)	< 0.001
RV Mass:Volume, g/ml	0.11 (0.09, 0.12)	0.12 (0.09, 0.14)	0.018

Variables are expressed as median (interquartile range). Group 1—patients with no interventions between serial CMRs; Group 2—patients with transcatheter or surgical interventions between CMRs. Median time between initial and latest CMR was 4.3 years (IQR 2.9, 6.6) for Group 1, and 2.7 years (IQR 1.6 to 5.3) for Group 2). *BSA* body surface area, *CMR* cardiovascular magnetic resonance, *EDVI* end-diastolic volume index, *ESVI* end-systolic volume index, *EF* ejection fraction, *PR* pulmonary regurgitation, *LGE* late gadolinium enhancement, *RV* right ventricle

**Table 3:** Mixed effects model of longitudinal changes

	Group 1 (n = 84)		Group 2 (n = 43)	
Variables	Mean 5 year change (95% CI)	*p*-value	Mean 5 year change (95% CI)	*p*-value
RV LGE score	− 0.88 (− 1.12, − 0.64)	< 0.001	− 0.54 (− 1.07, − 0.01)	0.047
% RV LGE extent	− 2.34 (− 2.91, − 1.76)	< 0.001	− 1.92 (− 3.18, − 0.67)	0.004
RVEDVI (ml/m^2^)	1.63 (− 2.37, 5.63)	0.421	− 42.2 (− 60.0, − 24.4)	< 0.001
RVESVI (ml/m^2^)	4.07 (1.15, 6.98)	0.007	− 19.1 (− 28.9, − 9.27)	< 0.001
RVEF (%)	− 2.81 (− 3.92, − 1.71)	< 0.001	− 3.11 (− 5.97, − 0.26)	0.033
PR fraction (%)	1.36 (− 0.04, 2.76)	0.057	− 24.1 (− 33.4, − 14.9)	< 0.001
RV Mass/BSA, g/m^2^	− 0.28 (− 1.03, 0.48)	0.470	− 3.46 (− 5.53, − 1.39)	0.002
RV Mass:Volume, g/ml	− 0.004 (− 0.01, 0.03)	0.243	0.01 (− 0.006, 0.03)	0.212

*p*-values < 0.05 denote that the slope of longitudinal change differs significantly from zero. Group 1—patients with no interventions between serial CMRs; Group 2—patients with transcatheter or surgical interventions between CMRs. *BSA* body surface area, *CMR* cardiovascular magnetic resonance, *EDVI* end-diastolic volume index, *ESVI* end-systolic volume index, *EF* ejection fraction, *PR* pulmonary regurgitation, *LGE* late gadolinium enhancement, *RV* right ventricle

### Comparison between groups

RV LGE score and % RV LGE extent showed a similar serial trend of gradual decline over serial CMRs in both Groups 1 and 2, with similar 5 yeaar rates of decline (Table 3). As seen in Fig. 1, in Group 1, no patient showed a serial increase in % RV LGE extent, while a small minority showed a slight increase in the RV LGE score. In Group 2, although the overall trend was similar to that in Group 1, a somewhat larger number of patients showed an increase in % RV LGE extent and RV LGE score. In this group, the only factor associated with an interval increase in both % RV LGE extent and LGE score (n = 6) was older age at repair (*p* = 0.009, supplemental Table 1).

### Factors associated with decline in RV LGE

We explored the hypothesis that a serial increase in RV end-diastolic volume indexed to body surface area (RVEDVI) with a corresponding increase in RV mass may lead to a serial decline in RV LGE score or % RV LGE extent. However, we found no significant association between the rate of change in either parameter of RV LGE, and the rate of change in RVEDVI.

## Discussion

In this serial CMR study of children and adults with repaired TOF, although RV LGE was present in all subjects on the initial CMR, the extent of RV LGE analyzed using 2 measures (LGE score and % LGE extent) did not significantly increase during follow-up, rather showing a slight decline over serial examinations.

### RV LGE—patterns and serial changes

The patterns of RV LGE in our cohort were similar to those previously reported, with the most common site for LGE being the RV outflow tract. [6] Several prior studies have identified the presence and extent of RV LGE as a risk factor for adverse long-term outcomes in TOF patients [2, 12, 13, 22]. However, the serial rate of change in the RV LGE extent has not been previously studied. Ylitalo et al. [23]. found in a cross-sectional study that the extent of RV LGE is higher in patients with a longer duration of follow-up and they hypothesized that LGE extent increased over time. However, the results of our longitudinal study suggest a lack of significant progress in RV LGE extent during a median follow-up period of 4 years. Further, we found no correlation between the extent of RV LGE and changes in RV volumes during follow-up. It is possible that the finding of a higher LGE extent in patients with a longer duration of follow-up in prior studies may reflect a higher extent of RV surgical scarring, possibly related to an older surgical era. The strengths of the current analysis are its longitudinal design and the quantitative assessment of RV LGE using 2 methods by a single observer.

### Comparison between groups

In the present analysis, we evaluated 2 groups of patients—those free of intervention between serial CMRs and those who had surgical or transcatheter intervention between serial CMRs. We analyzed these groups separately, since surgical manipulation of the outflow tract, as well as artifact caused by implanted valves could potentially confound the serial assessment of LGE extent. The findings of a similar lack of progression in RV LGE in both groups suggests that RV LGE in TOF patient possibly represents fixed post-surgical scarring that is not significantly modulated by long term volume loading due to pulmonary regurgitation, or by intervening procedures, at least in the intermediate term (median follow-up duration was 4 years).

### LGE assessment

There are inherent difficulties in quantifying RV LGE due to the thin free wall. We used a previously described method for calculation a semiquantitative RV LGE score, in addition to a new method to quantify RV LGE extent [8]. In using the second method for quantifying RV LGE extent, we avoided techniques that automatically designate areas of LGE using a pre-defined standard deviation cutoff relative to normal myocardium, as this can be fraught with error in the thin-walled RV. Instead, we manually identified areas of RV LGE and then quantified the extent of RV LGE in these areas. We found good correlation in the extent of RV LGE calculated using both methods and longitudinal trends in RV LGE were also similar using both techniques. We found good inter-rater and modest inter-observer reliability in quantifying % RV LGE extent. Good inter-rater reliability in assessing the RV LGE score has been previously reported [8].

### Clinical implications

Our results have important clinical implications. Since we did not observe a significant increase in RV LGE over a median follow-up duration of 4 years, this suggest that RV LGE does not progress rapidly. Therefore, frequent serial assessment of LGE may not be necessary, thereby avoiding the frequent administration of gadolinium-based contrast agents in these patients, in light of several reports of long-term retention of gadolinium in patients who receive multiple doses of contrast [17, 18]. It should be noted that our findings apply to patients in a more recent surgical era.

## Limitations

Several limitations of the current work are worth considering. First, there are known limitations related to its retrospective single-center design. However, these are likely offset by the use of serial longitudinal data using a consistent imaging protocol and analysis by a single observer. Second, while we used serial CMRs, the median duration of follow-up was 4 years and therefore we are not able to exclude progression in RV LGE over longer periods of time. Studies with larger numbers of patients with a longer duration of follow-up may help answer this question more definitively. Third, due to its retrospective design, we did not have high resolution 3-D LGE imaging or imaging for diffuse fibrosis available for serial assessment, as these techniques have only become available more recently. It is possible that follow-up studies using these more sensitive techniques may detect progression in RV fibrosis. Finally, we did not have sufficient numbers of subjects with LV LGE to analyze longitudinal changes in LV LGE over time.

## Conclusions

In children and adults with repaired TOF, the RV LGE extent does not increase significantly based upon serial CMRs during a median follow-up of 4 years. Given recent concerns regarding long-term gadolinium retention, our data do not support the routine frequent serial assessment of LGE in TOF patients.

## Data Availability

The datasets generated and/or analysed during the current study are not publicly available, as this was not approved by the hospital’s Committee on Clinical Investigation for this study.

## Abbreviations

CMR: Cardiovascular magnetic resonance
ICC: Intraclass correlation coefficient
IQR: Interquartile range
LGE: Late gadolinium enhancement
PA: Pulmonary artery
PR: Pulmonary regurgitation
RV: Right ventricle/right ventricular
RVEDVI: Right ventricular end-diastolic volume index
RVEF: Right ventricular ejection fraction
RVESVI: Right ventricular end-systolic volume index
TOF: Tetralogy of Fallot
